# Magnetic Resonance Imaging of Tumors Colonized with Bacterial Ferritin-Expressing *Escherichia coli*

**DOI:** 10.1371/journal.pone.0025409

**Published:** 2011-10-03

**Authors:** Philip J. Hill, Jochen Stritzker, Miriam Scadeng, Ulrike Geissinger, Daniel Haddad, Thomas C. Basse-Lüsebrink, Uwe Gbureck, Peter Jakob, Aladar A. Szalay

**Affiliations:** 1 Genelux Corporation, San Diego, California, United States of America; 2 School of Biosciences, University of Nottingham, Leicester, United Kingdom; 3 Virchow Center, Biomedical Research, School of Medicine, University of Würzburg, Würzburg, Germany; 4 Institute of Biochemistry, Biocenter, Würzburg, Germany; 5 Institute for Molecular Infection Biology, Würzburg, Germany; 6 Institute for Experimental Physics V and MRB Research Center, Würzburg, Germany; 7 University of California San Diego Center for Functional MRI, La Jolla, California, United States of America; 8 Department of Functional Materials in Medicine and Dentistry, University of Würzburg, Würzburg, Germany; City of Hope National Medical Center and Beckman Research Institute, United States of America

## Abstract

**Background:**

Recent studies have shown that human ferritin can be used as a reporter of gene expression for magnetic resonance imaging (MRI). Bacteria also encode three classes of ferritin-type molecules with iron accumulation properties.

**Methods and Findings:**

Here, we investigated whether these bacterial ferritins can also be used as MRI reporter genes and which of the bacterial ferritins is the most suitable reporter. Bacterial ferritins were overexpressed in probiotic *E. coli* Nissle 1917. Cultures of these bacteria were analyzed and those generating highest MRI contrast were further investigated in tumor bearing mice. Among members of three classes of bacterial ferritin tested, bacterioferritin showed the most promise as a reporter gene. Although all three proteins accumulated similar amounts of iron when overexpressed individually, bacterioferritin showed the highest contrast change. By site-directed mutagenesis we also show that the heme iron, a unique part of the bacterioferritin molecule, is not critical for MRI contrast change. Tumor-specific induction of bacterioferritin-expression in colonized tumors resulted in contrast changes within the bacteria-colonized tumors.

**Conclusions:**

Our data suggest that colonization and gene expression by live vectors expressing bacterioferritin can be monitored by MRI due to contrast changes.

## Introduction

Magnetic Resonance Imaging (MRI) has revolutionized *in vivo* imaging due to its non-invasive nature, high spatial resolution and extraordinary tissue contrast abilities.

The ability to non-invasively monitor gene expression *in vivo* in a longitudinal fashion further expands the utility of MRI as a tool in both basic research and ultimately in the clinical setting. For example, the development of reporter gene technologies that provide a readily measurable signal of transcriptional activity enable non-destructive gene expression studies during developmental or disease processes.

Many bacterial strains tested show preferential replication in solid tumors. Using those replication capabilities to develop cancer therapies and/or diagnostics has become an area of growing interest in recent years (see [1], [2], [3], [4], [5] for reviews). Among these are anaerobic bacteria like *Bifidobacteria* or *Clostridia*, whose spores germinate in the anoxic/hypoxic regions of tumor tissues prior to replication and show promising results in tumor therapy. In addition to these strict anaerobes, facultative anaerobic bacteria have also been shown to replicate inside solid tumors. Most of the bacteria used in these studies were derived from pathogenic bacteria. In several studies, we and others recently showed that probiotic *E. coli* Nissle 1917 (*Ec*N) is also able to colonize and exclusively replicate in tumor tissue [6], [7], [8], [9]. In contrast to most tumor-targeting bacteria, probiotic bacteria do not replicate in organs (e.g., spleen and liver), perhaps due to the lack of virulence factors in this strain and lead to ratios exceeding 10^6^∶1 of bacteria in the tumor compared to bacteria in specific organs.

The specificity of tumor-colonizing bacteria makes them suitable as simultaneous therapeutic and diagnostic agents for cancer. Therapeutic effects have already been demonstrated using *Clostridia* and *Salmonella* strains [10], [11]. Moreover, when such bacteria are engineered to express therapeutic genes (e.g. toxins, prodrug converting enzymes, therapeutic siRNAs, immunmodulatory molecules) they can elicit enhanced therapeutic effects (reviewed in [12]).

Therefore, for future clinical studies, it will be crucial to reliably detect and monitor the presence of bacteria in tumors (and in deep tissues) without needing to excise the respective tissues. So far, non-invasive optical fluorescence and/or low light imaging [6], [13], [14], [15], as well as SPECT or PET imaging [16], [17], [18] have been used to visualize the presence and distribution of bacteria within tumor-bearing mice. The current optical imaging modalities are, however, limited by light scattering and light extinction in tissues and are, therefore, mainly limited to small animal imaging or near-surface detection. In contrast, radiotracer imaging (e.g., PET) is not limited by tissue depth but shows poor spatial and anatomical resolution. MRI, on the other hand, has the potential to provide much better resolution, but is generally less sensitive.

Ferritins are ubiquitous iron storage proteins found in species ranging from microbes to man [19], [20]. Their function is to maintain a cellular reservoir of iron in a non-toxic, bioavailable form, and to protect the host cell from oxygen and its free radical products [21], [22]. This is achieved via a ferroxidase function that converts Fe^2+^ to Fe^3+^, the paramagnetic form of iron responsible for *T2* contrast modification in MRI. In MRI transverse relaxation (T2) is shortened by the presence of iron and its reciprocal, known as *R2* is directly proportional to iron concentration. Expression of human H-chain ferritin can be used to generate contrast in T2 weighted MRI *in vivo* [23], [24], [25], [26], [27], [28], [29], [30], [31], causing a local reduction in the signal intensity. The iron loading of human ferritin, which has been intensively studied at different magnetic field strengths, results in changes to the longitudinal (*R*_1_ = 1/*T*_1_) and transverse (*R*_2_ = 1/*T*_2_) relaxation rates of water [32], [33]. It has been shown that human ferritin creates a unique linear dependence of *R_2_* on the magnetic field [34], [35].

In bacteria, three types of ferritin-like proteins are recognized: the archetypal ferritins (e.g., *ftn*), the heme-containing bacterioferritins (e.g., *bfr*), and the smaller Dps-type ferritins (e.g., *fri* of *Listeria*). All of these peptides are capable of forming endogenous nanoparticles consisting of a protein shell surrounding a hydrated iron oxide core of up to 4,500 iron atoms [22].

Three-dimensional structures have been deduced for human H-chain, horse spleen, rat liver, *Escherichia coli*, and bullfrog ferritins, as well as for the bacterioferritin (Bfr) of *E. coli*, demonstrating high homology between the different ferritin-like proteins [36], [37], [38], [39], [40]. The ferroxidase activity of mammalian ferritins is due to an active site (the ferroxidase center) in the middle of the four-helix bundle of each H-chain subunit where a dinuclear iron species is thought to form [36], [41]. Key amino acid residues in the ferroxidase centers of ferritins are conserved in bacterioferritins, suggesting that Bfr possesses a ferroxidase center similar to that of H-chain ferritins [42], [43]. This suggestion has been confirmed by studies on iron uptake by wild-type Bfr and site-directed mutants of Bfr [44], and is also supported by the presence of a dinuclear metal-binding site at the ferroxidase center in the Bfr crystal structure [40].

The presence of at least seven iron uptake systems in *E. coli* Nissle 1917 [45], [46] makes this strain an attractive candidate for iron acquisition. Here, we investigate the feasibility of using three different bacterial ferritins as reporter genes in *Ec*N for magnetic resonance imaging in animal studies. We demonstrate that tumors colonized by *Ec*N can be visualized and detected based on enhanced iron accumulation due to bacterial amplification, iron uptake, and iron storage upon exogenous induction using L-arabinose as a non-toxic inducer of gene expression.

## Results

### Iron accumulation in *Ec*N over-expressing ferritin-like proteins

**Figure 1:**
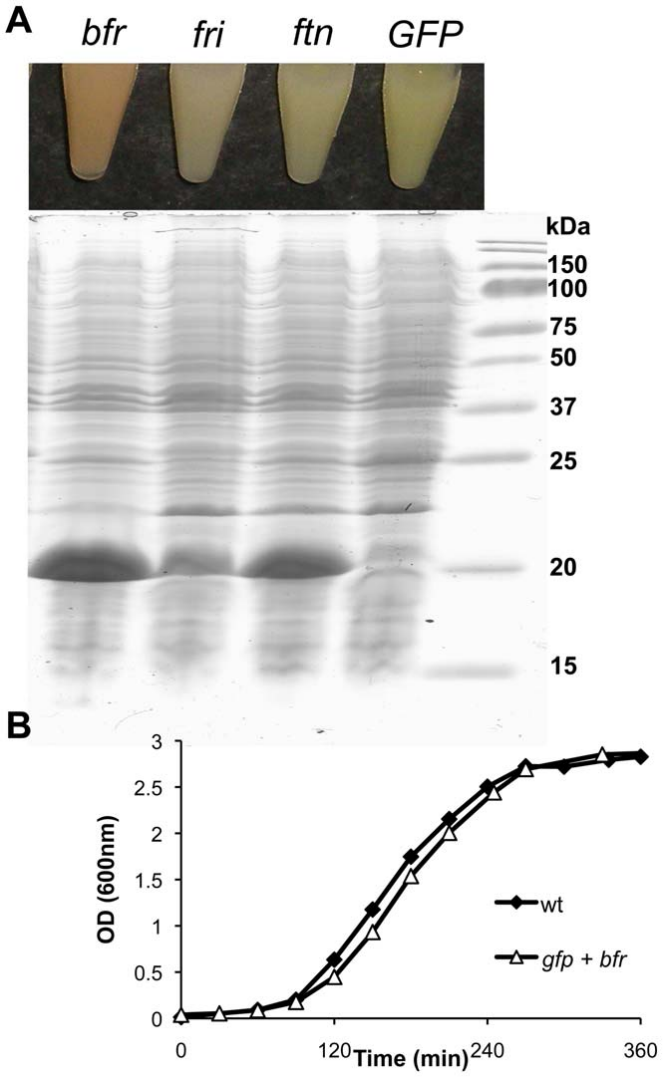
Expression of various bacterial ferritin-like proteins in *Ec*N. **A**) The over-expression of heme containing Bfr results in a color change of the bacteria (upper picture, left tube). The overexpression of Bfr (18.4 kDa), Fri (18.0 kDa), and Ftn (19.3 kDa) was confirmed by Coomassie-stained SDS-PAGE gels. **B**) Only minimal effects on bacterial growth were observed when wild-type (wt) *Ec*N or *GFP*- and *bfr*-encoding *Ec*N (*gfp* + *bfr*) were grown in BHI. Standard deviation (<2%) of measured values is not shown.

We evalutated different bacterial ferritin-like proteins as MRI reporter genes. We, therefore, overexpressed the genes for three different ferritins (*E. coli* ferritin-*ftn*; *E. coli* bacterioferritin-*bfr*; and *L. monocytogenes* non-heme iron-binding protein-*fri*) in *E. coli* Nissle 1917. To determine whether expression of additional iron storage proteins alone leads to increased iron accumulation in the bacteria, we chose to overexpress *L. monocytognes fri* instead of the structurally related *E. coli dps*. In contrast to Dps, Fri does not have DNA-binding capacity and, therefore, will not directly affect expression of other genes. Expression of GFP served as a negative control. Each of these genes was initially placed under control of the constitutive *Bacillus megaterium xylA* promoter (P*_xylA_*), which resulted in elevated expression of the target genes in *Ec*N (Fig. 1A). The over-expression of the heme-containing Bfr resulted in a color change of the bacteria (to red) and the presence of the iron storage proteins was confirmed by Coomassie-staining of SDS-PAGE gels.

Even though the amount of Bfr reaches up to 35% of the total bacterial protein content, the effect on the bacterial growth (evaluated by optical density measurements at 600 nm) of the GFP and Bfr expressing strain was negligible (Fig. 1B).

**Figure 2:**
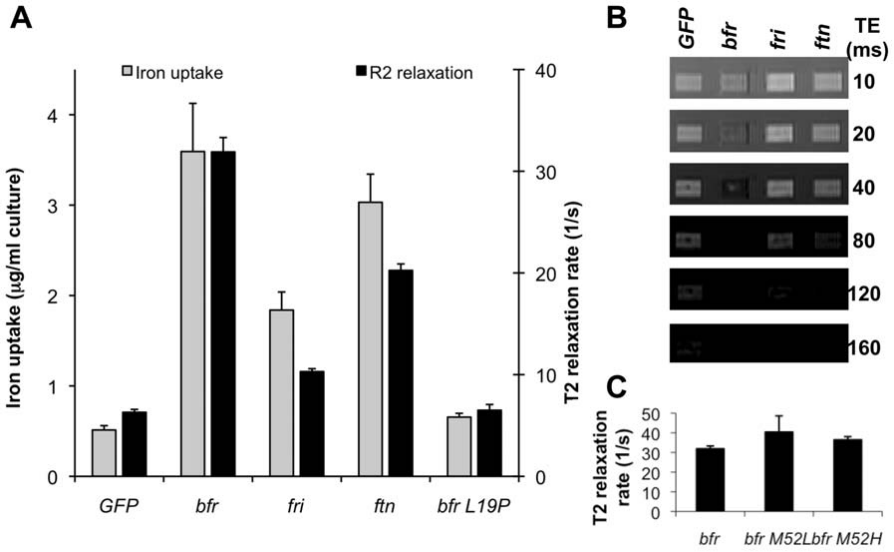
Effects of ferritin-like proteins on iron uptake and R2 relaxation. **A**) Iron uptake and the effects on *R2* relaxation were determined. Data represent mean (n = 3) plus standard deviation of one representative experiment. Expression of Bfr resulted in the highest iron uptake and the most obvious effects on *R2* relaxation. **B**) Bacterial suspensions were put into wells of agarose-gels and images were obtained using a TR of 4000 ms with varying TEs to determine the *R2* relaxation values in **A**). **C**) Expression of non-heme containing Bfr did not result in decreased *R2* relaxation (p>0.05).

Overnight growth of ferritin-expressing bacteria in iron-supplemented brain heart infusion (BHI) broth resulted in augmented iron accumulation in *Ec*N cells. This was shown by quantitative inductively coupled plasma mass spectrometry (ICP-MS) analysis performed on bacterial lysates treated with Proteinase K to release iron atoms from the iron-storage proteins (Fig. 2A). The results indicate that increased iron accumulation occured upon overexpression of all the ferritin-like proteins but that the level of iron accumulation was dependent on the type of ferritin-like protein used. Cultures of Bfr-expressing *Ec*N cells showed a red coloration due to high amounts of heme-containing Bfr (Fig. 1) and stored similar amounts of iron when compared to Ftn- (p = 0.21), but more than Fri- (p<0.01) or GFP-control bacteria (p<0.01). Ftn- and Fri-expressing *Ec*N cells showed 5.9- and 3.6-fold more iron storage than control bacteria (Fig. 2A, grey bars, p<0.01).

Quantitative analysis of Coomassie-stained protein gels enabled calculation of the number of ferritin molecules and indirectly facilitated calculation of the average number of iron atoms per molecule of ferritin, using the ICP-MS data. Ftn was found to load the highest number of iron atoms (approx. 75 per holomer), followed by Bfr (approx. 44 iron atoms per holomer). Fri bound the lowest amount of iron atoms (approx. 10 per holomer).

### Effects of iron-storage protein expression levels on *R2* relaxation rates

In addition to the iron determination experiments, samples were prepared for *R2* relaxation measurements. Bacteria were harvested from overnight cultures grown in BHI supplemented with ferrous citrate, washed with phosphate-buffered saline (PBS) and re-suspended in a 1∶1 mix of PBS and OCT embedding medium to prevent bacteria from sedimenting during T2 relaxation rate measurements. Samples were analyzed in a 7T small animal MRI scanner using a spin echo sequence and *R2* relaxation rates were determined (Fig. 2A derived from values obtained from images in Fig. 2B). All ferritin overexpressing strains showed a significant increase in transverse relaxation compared to control cultures (p<0.01). Bfr overexpression resulted in the highest negative contrast, followed by Ftn and then Fri (p≤0.01 for all possible comparisons). As Bfr overexpressing *Ec*N accumulated most iron and also showed highest impact on *R2* relaxation (see below), all mouse experiments were performed with *bfr*-encoding *Ec*N.

### Enhanced *R2* relaxation by Bfr

When diluting Bfr expressing bacteria in wild-type *Ec*N (1∶1 and 1∶3 mix, respectively), the *R2* relaxation decreased accordingly (R^2^-value: 0.9759) as shown in Figure S1. The concentration of bacteria in the agarose gels corresponded to a 40-fold concentrated over-night culture of 2−3×10^9^ cfu/ml resulting in about 1×10^11^ cfu/ml. Therefore, 1×10^11^ cfu of the 100% pure Bfr-expressing *Ec*N culture resulted in a T2 relaxation rate of approx. 30, 50% in a T2 relaxation rate of approx. 17 etc.

Furthermore, we found that Bfr had a 50% greater effect upon *R2* relaxation than Ftn, even though iron levels in the samples were almost equivalent. Therefore, we decided to investigate whether Bfr was the most suitable reporter, due to the presence of heme iron on the Bfr molecule. Ferritin-like proteins isolated from bacteria may be free of heme or may contain heme, with bacterioferritins containing up to 12 heme molecules per holomer [21].

To determine the effect of the Bfr hemes on *R2* relaxivity, we generated Bfr mutants in which the heme binding methionine residue (Met52) [40] was replaced using site-directed mutagenesis with either a leucine (Bfr M52L) or a histidine (Bfr M52H). These mutations resulted in a Bfr lacking heme groups [44]. Furthermore, an additional point mutation L19P was generated to disrupt the first alpha helix of Bfr, resulting in Bfr-expressing strains that did not accumulate additional iron (Fig. 2A).

The lack of heme binding capacity in Bfr M52L and Bfr M52H was readily observed when the bacteria were grown in iron-supplemented BHI overnight. While the Bfr-overexpressing strain showed the typical red color of heme, the heme-negative mutants appeared like wild-type *Ec*N. Nevertheless, the iron uptake and storage was not negatively affected and *R2* relaxation rates were not reduced in the absence of heme in Bfr M52L and Bfr M52H (Fig. 2C). However, effects on *R2* relaxation and iron storage were abrogated when the helix-disrupting L19P mutation was introduced (p<0.01) (Fig. 2A).

### Induction of bacterioferritin in murine tumors colonized by *Ec*N

**Figure 3:**
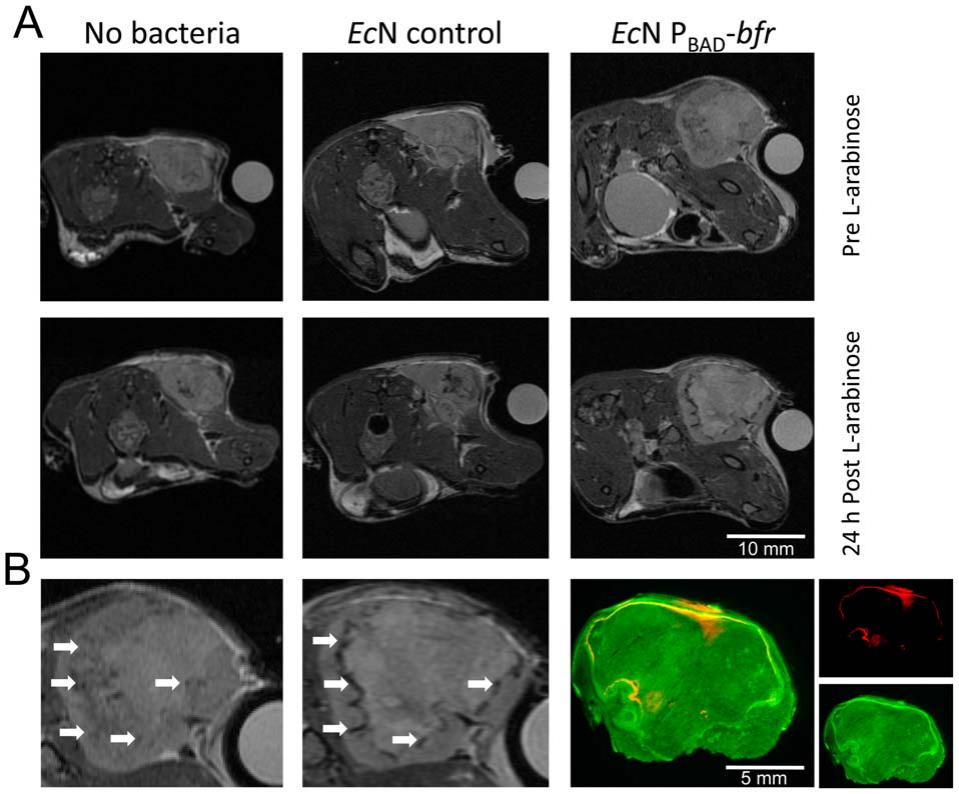
Effects of induced Bfr expression on T2 weighted imaging *in vivo*. **A**) Tumor-bearing mice were injected with PBS (no bacteria), an *Ec*N control strain (*Ec*N control), or *Ec*N expressing Bfr upon induction with L-arabinose (*Ec*N P_BAD_-*bfr*). Mice were imaged just before, and 24 h after injection of L-arabionse. Only in the Bfr overproducing *Ec*N were distinct dark regions visible 24 h after the inducer was injected. **B**) Enlarged *Ec*N P_BAD_-*bfr* colonized tumor before and 24 h after L-arabinose injection. Arrows point to the dark rim around the necrotic center of the tumor corresponding to immunofluorescent staining of *Ec*N (red) in sections of (a different) tumor (right panels). Actin was stained green.

We recently reported the use of L-arabinose as an exogenous inducer for gene expression in tumor-colonizing *Ec*N [6]. *Ec*N cells carrying a plasmid with the *luxABCDE*-operon of *Photorhabdus luminescens*, under control of the P_BAD_-promoter, were injected into tumor-bearing mice. Light emission from *Ec*N-colonized tumors was observed for about 24 h after systemic administration of L-arabinose, while no light was detected in the absence of L-arabinose. Here, we demonstrated that Bfr-carrying *Ec*N colonize tumors equally well compared to wild-type *Ec*N (data not shown) and used the same L-arabinose dependent promoter system for induction of *bfr* expression, which was the most promising as a MRI reporter gene. MRI was performed in a 7T small animal scanner (echo time (TE)/repetition time (TR): 30/4000 ms) before injection and after 1 h, and 24 h following intraperitoneal (IP) injection of L-arabinose. One hour after L-arabinose administration, the tumors revealed non-specific darkening around the tumor vasculature by MRI, compared to images of the same animals taken before injection of the inducer (data not shown). This darkening was probably due to osmotic effects caused by the inducer compound. Twenty four hours after inducer injection, the non-specific ‘L-arabinose-effect’ was no longer apparent. Further, a significant contrast increase was observed in tumors colonized with bacteria carrying plasmid DNA with the *bfr* gene under control of the inducible P_BAD_ promoter (see Figure S2 for a 3D-reconstruction). This high contrast zone, lining the necrotic center of the tumor, was not observed in non-colonized tumors or in tumors colonized with a control *Ec*N strain expressing GFP rather than Bfr (Fig. 3A). Furthermore, the dark zone strongly resembled the bacterial distribution within the tumor, as verified by independent immunofluorescent staining of 100 µm sections of another tumor which also was colonized with the *bfr* encoding *Ec*N (Fig. 3B).

### Enhanced *R2* relaxation in *Ec*N-colonized tumors after *bfr* induction

**Figure 4:**
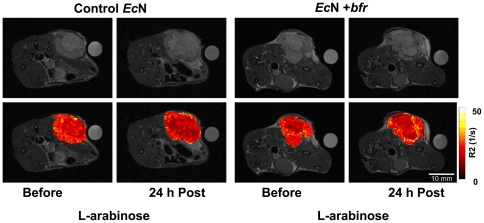
*R2* relaxation maps of tumors obtained before and after induction of protein expression. Tumor-bearing mice were injected with control bacteria (left) or P_BAD_-*bfr* encoding *EcN* (right). Upper panels: Before, and 24 h after injection of L-arabinose, mice were imaged in a 7T small animal scanner (TE/TR 30/4000 ms:). Lower panels: In addition, images at TE: 10, 20, 40, and 80 ms (TR: 4000 ms) were obtained and used to generate *R2* relaxation maps of tumors, which were overlayed on top of the images shown in the upper panels.

We also measured the changes on *R2* relaxation 24 h after induction of *bfr* expression in colonized tumors (Fig. 4). R2 relaxation maps were obtained from images of mice that were scanned with constant TR (4000 ms) but varying TE (10, 20, 40 and 80 ms). While no significant changes were observed in control tumors, the *R2* relaxation around the necrotic region of tumors colonized with the *bfr*-encoding *E. coli* Nissle 1917 increased dramatically and was comparable to *R2* relaxation rates obtained in overnight cultures (Fig. 2A and Fig. 1).

In the tumors the concentration of bacteria was about 1−2×10^9^ cfu/g. In the tumor sections (e.g., Fig. 3B), the bacteria were concentrated in a small area within the tumor mass (about 5% of the total tumor area in Fig. 3B resulting in a local concentration of approx. 3×10^10^ cfu/g). Furthermore, the bacterial concentration within the bacteria-colonized area was not evenly distributed. Therefore, the concentration of bacteria reached similar levels as those used in the agarose gels (1×10^11^ cfu/ml) which explains the similar *R2*-values.

## Discussion

The iron storage properties of bacterial ferritin-like proteins have been known for years, yet no detailed investigations have been carried out to ascertain whether they are suitable as MRI reporter genes *in vivo*. Recent studies successfully tested the potential of magnetotactic bacteria (*Magnetospirillum magneticum*) or one of their genes (*magA*) for the detection of tumors [47], [48], as well as the human H-chain ferritin as a reporters for non-invasive MRI of mammalian cells in different applications [23], [24], [25], [26], [27], [28], [29], [30], [31]. Here we report, for the first time, the evaluation of bacterial ferritin-like genes (namely *bfr*, *fri*, and *ftn*) as MRI reporter genes expressed in prokaryotic organisms. We showed that overexpression of bacterial ferritin-like proteins, in general, can be used to significantly affect transverse relaxation in MRI.

When overexpressing the iron-storage proteins, we determined that the iron content of the bacteria significantly increased (up to 6-fold in Bfr-expressing *Ec*N in culture), which is consistent with previous reports on their role as iron-storage proteins. Also, the average number of iron atoms we found per holomer were comparable to earlier reports [49], although we did detect more iron ions in Ftn (75, in contrast to 5–20). These findings might be due to the different growth conditions used or to the fact that more iron storage proteins were expressed in this system (about 35% and 16% of total protein were Bfr and Ftn, respectively, while previously 3–18% and 11–14% were reported [49]).

The most striking structural difference between bacterioferritin and the other ferritin-like proteins is the presence of heme, with up to 12 molecules bound to one 24-mer of Bfr. However, we found that heme-free Bfr-mutants still exhibited the same effects on *R2* relaxation rates as wild-type Bfr. Therefore, the presence of heme in Bfr alone is not responsible for the enhanced effect of Bfr on *R2* relaxation, compared to other ferritin-like reporters. Differences in the intrinsic mechanisms of iron packaging within the protein shell, even at similar iron concentrations, or the activity of the respective ferroxidase center could account for these findings. The differences in iron load might also explain the effects on *R2* relaxation, since changes in iron contents within human ferritin was previously reported to affect longitudinal and transverse relaxation rates [33]. Differences in the structure (e.g. Bfr and Ftn are built of 24-mers, while Fri complexes are made out of 12 monomers) or in the maximal iron uptake capacity (Bfr and Ftn: 2000–3000, and Dps-like 12-mers like Fri: 500 Fe molecules/holomer [50]) did not seem to have marked influence on the generated contrast. Nevertheless, these studies showed that Bfr has the strongest effect on *R2* relaxation under the experimental conditions used.

Induction of Bfr in *Ec*N-colonized tumors provided unequivocal evidence for the utility of the *bfr* gene as a reporter. In the tumor regions colonized by *Ec*N, we saw significant effects on contrast in T2-weighted images 24 h after administration of the inducer L-arabinose. The contrast change very strongly resembled the characteristic distribution of *E. coli* in tumors that could be observed in Fig. 3B as well as in previous publications by us and others [6], [8], [51], [52]. The yet unexplained L-arabinose effect early after administration, maybe due to osmotic effects resulting in unspecific darkening in some regions of the tumor. Furthermore, unspecific darkening was observed in the central region of some tumors (e.g., in the control tumors in Fig. 3) but these do not correlate with the bacterial distribution and are also visible in non-colonized tumors. Despite those effects, we are convinced that expression of Bfr offers great utility in MRI-based visualization of bacterial infections, as well as for colonization of tumors in deep tissues. Alternatively, constitutive overexpression of Bfr could provide a marker protein to track the movement of bacteria in complex systems by MRI, for example, in non-invasively monitoring bacterial distribution within tissues. Moreover, tumor-colonizing bacteria expressing *bfr* or similar genes under control of externally activated promoters could be used to efficiently locate tumors and metastases by comparing images generated pre- and post- gene induction and during cancer therapy. Also, it is likely that the described approach will be applicable to the study of intercranial as well as peripheral tumors given that L-arabinose can readily cross the blood brain barrier [53], [54]. The L-arabinose uptake in the brain has been shown to be competitively inhibited by glucose, suggesting that the transfer to the brain is mediated by glucose transporters as has been reported for similar transporters from erythrocytes and adipose tissue [55], [56]. In contrast to the recently described PET-imaging approach taking advantage of the endogenously expressed *E. coli* thymidine-kinase [18], the described system lacks the potential to quantify the number of bacteria within the tumor tissue and is dependent on the overexpression of bacterioferritin. Therefore, it cannot be used for imaging natural infections. However, the spatial resolution using MRI is much better when compared to PET. In addition, the L-arabinose inducible system allows imaging before and after contrast change which is not possible with the described PET-procedure. Therefore, both systems do have their advantages and limitations thus making a combination of both modalities desirable.

The use of ferritin-like molecules as reporter proteins may affect the iron homeostasis in the host organism. High levels of ferritin in the cytoplasm could potentially reduce the amount of free Fe^2+^ ions and, therefore, derepress iron uptake systems. In bacteria, this could result in the induction of siderophore biosynthesis and other iron-scavenging systems such as heme and transferrin-binding proteins. However, in most bacteria, when inside a mammalian host, the low free-iron availability acts as a signal to upregulate these systems. Moreover, any augmentation of iron uptake is likely to result in increased iron storage, which could increase the sensitivity of the Bfr reporter system. In these studies, we observed only minor differences in bacterial growth in cultures (Fig. 1B) and no differences in colonization of tumors between *Ec*N and its derivatives (data not shown), which express either GFP alone or Bfr and GFP together. This indicates that Bfr expression has no detrimental effect on the bacterial physiology.

In conclusion, we propose that the use of bacterioferritin expression is not limited to tumor detection using bacteria alone. Although not experimentally tested, we think that *bfr*, carried by oncolytic viruses, would also enable the clinical monitoring of therapeutic efficacy of these biological therapies. However, the iron uptake into the virus infected cells must be guaranteed (e.g., by co-expression of a transferrin receptor). The expression of bacterioferritin in pathogenic organisms could also help to analyze and characterize infection processes in deep tissues of live (larger) animals in real time with high spatial resolution. Furthermore, the bacterioferritin-expressing live vectors could be equipped with additional reporter genes (e.g., luciferases or PET reporter genes), enabling multi-modality imaging approaches that combine the high spatial resolution of MRI with the high sensitivity of optical and/or PET imaging.

## Materials and Methods

### Ethics statement

All animal experiments were carried out in accordance with protocols approved by the Institutional Animal Care and Use Committee (IACUC) of Explora BIOLABS, located in San Diego Science Center (San Diego, USA) (protocol number: EB08-003), the IACUC of the University of California, San Diego (San Diego, USA) (protocol numbers: R06041 and R08335), and/or the “Regierung von Unterfranken” (Würzburg, Germany) (protocol number AZ 55.2-2531.01-17/08).

### Bacterial strains and plasmids

Plasmid-cured *E. coli* Nissle 1917 (*Ec*N) was transformed with plasmids indicated in Table 1. Primer sequences for PCRs used to construct the plasmids can be found in Table S1.

**Table 1:** Plasmids used in this study.

Name	Relevant Properties	Reference or Source
pENTR-P_BAD_	P*_araBAD_*, *araC,* kan^R^, *attL4*, *attR1*	(6)
pENTR-P*_xylA_*	P*_xylA_*, kan^R^, *attL4*, *attR1*	T. Perehinec
pDONR221	*attP1*, *attP2*, kan^R^, *ccdB*, cm^R^	Invitrogen
pENTR221-*GFP*	*GFP*, kan^R^, *attL1*, *attL2*	T. Perehinec
pENTR-*bfr*	*bfr*, kan^R^, *attL1*, *attL2*	This work
pENTR-*bfr*_M52L_	*bfr*_M52L_, kan^R^, *attL1*, *attL2*	This work
pENTR-*bfr*_M52H_	*bfr*_M52H_, kan^R^, *attL1*, *attL2*	This work
pENTR-*bfr*_L19P_	*bfr*_L19P_, kan^R^, *attL1*, *attL2*	This work
pENTR-*ftn*	*ftn*, kan^R^, *attL1*, *attL2*	This work
pENTR-*fri*	*fri*, kan^R^, *attL1*, *attL2*	This work
pENTR-term	*rrnB*T1T2, kan^R^, *attL3*, *attR2*	(6)
pENTR2RP3-GFP	*GFP*, kan^R^, *attL3*, *attR2*	T. Perehinec
pBR322DEST	*rop, ccdB*, cm^R^, amp^R^_,_ tet^R^, *attR3*, *attR4*	(6)
pBR322DEST_inv_	*rop, ccdB*, cm^R^, amp^R^_,_ tet^R^, *attR4*, *attR3*	This work
pBR322DEST_inv_-P*_xylA_*-*GFP*-term	P*_xylA_, GFP, rrnB*T1T2, *rop,* amp^R^_,_ tet^R^	This work
pBR322DEST_inv_-P*_xylA_*-*bfr*-GFP	P*_xylA_, bfr*, *GFP, rop,* amp^R^_,_ tet^R^	This work
pBR322DEST_inv_-P*_xylA_*-*ftn*-*GFP*	P*_xylA_, ftn*, *GFP, rop,* amp^R^_,_ tet^R^	This work
pBR322DEST_inv_-P*_xylA_*-*fri*-*GFP*	P*_xylA_, fri*, *GFP, rop,* amp^R^_,_ tet^R^	This work
pBR322DEST-P*_BAD_*-*bfr*-GFP	P*_BAD_, bfr*, *GFP, rop,* amp^R^_,_ tet^R^	This work

For construction of pBR322DEST_(inv)_, the *ccdB* gene for negative selection and the chloramphenicol resistance gene *cm^R^* for counterselection, flanked by *attR3* and *attR4* recombination sites, were PCR amplified from pDESTR4-R3 using ccdBf ClaI and ccdBr ClaI primers, digested with *Cla*I, ligated with *Cla*I restricted and calf intestinal alkaline phosphatase treated pBR322. The mixture was then transformed into *E. coli ccdB* Survival T1^R^. Orientation of the insert was confirmed by *Pst*I digest and agarose gel electrophoresis. In pBR322DEST, *attR4* is closer to the β-Lactamase of pBR322 and, in pBR322DEST_inv_, the *attR3* site is closer.

The ferritin entry-clone plasmids pENTR-*bfr*, pENTR-*ftn*, and pENTR-*fri* were constructed as follows: Each of the three open reading frames was PCR-amplified with the appropriate attB1F and attB2R primers, using genomic DNA (*Ec*N for *bfr* and *ftn*; *L. monocytogenes* for *fri*) as a template, followed by a BP-recombination into pDONR221, according to the instructions of the manufacturer (Invitrogen, Carlsbad, USA). For construction of plasmids pENTR-*bfr*_M52L_, pENTR-*bfr*_M52H_, and pENTR-*bfr*_L19P_, point mutations were inserted using recombinant PCR. The 5′-end of the gene was PCR-amplified using pENTR-*bfr* as a template and primers bfr-attB1F and bfr-M52L-rev, bfr-M52H-rev, or bfr-L19P-rev while the 3′-end was amplified using bfr-attB2R and bfr-M52L-for, bfr-M52H-for, or bfr-L19P-for. In a second PCR, the 5′- and 3′-end products were mixed and bfr-attB1F and attB2R were used as primers. The resulting PCR fragment was used for a BP-recombination into pDONR221.

The plasmids pBR322DEST-P_BAD_-*bfr*-GFP, pBR322DEST_inv_-P*_xylA_*-*bfr*-GFP, pBR322DEST_inv_-P*_xylA_*-*ftn*-GFP, and pBR322DEST_inv_-P*_xylA_*-*fri*-GFP were obtained after 3-way LR-recombination performed as described in the Multisite Gateway® three vector construction kit protocol of the manufacturer (Invitrogen, Carlsbad, USA). We chose to use pBR322 as plasmid backbone as this plasmid was the most stable plasmid that we tested in *Ec*N both in culture as well as in tumor bearing mice (data not shown).

### NMR and MRI imaging of *E. coli* cells and *E. coli* colonized tumors

Twenty ml of overnight cultures of *Ec*N strains grown in BHI supplemented with 150 µM ferrous citrate were washed twice and resuspended in 20 ml PBS. During the second wash, the amount of bacteria per ml was adjusted so that each suspension had identical optical densities at 600 nm.

The pellet derived from this suspension was resuspended in 500 µl PBS/OCT (1:1 mixture), loaded into wells of an agarose gel (5% agarose in degassed water), and overlayed with 5% agarose in water. The gel was then imaged in a 7T small animal MRI machine (GE Medical Systems, Brooksville, Florida, USA) to calculate the T2 changes in the gels (TE/TR 10–160/4000 ms, Matrix 256×128 slice thickness 0.3 mm, 3 averages). Alternatively, 250 µl of the suspension was added to 0.3 ml PCR-tubes and analyzed (multi spin echo sequence with TE/TR 20–320/1400 ms, slice thickness 2 mm, 2 averages, matrix 256×256, and field of view 60×40 mm^2^) in a 7T small animal scanner (Bruker, Ettlingen, Germany).

For tumor models, BALB/c mice were obtained from Harlan (Indianapolis, Indiana, or Borchen, Germany). Five- to six-week-old female mice were injected s.c. with 3.3×10^4^ murine 4T1 mammary cancer cells (ATCC: CRL-2539) re-suspended in 100 µl PBS. The authentication of cells were analyzed by microscopy and growth in immunocompetent BALB/c mice. For colonization studies, bacteria were grown in 5 ml of LB-broth containing 100 µg ml^−1^ ampicillin to an optical density of 0.4 at 600 nm (approx. 2×10^8^ CFU/ml). The cells were washed twice in PBS, then diluted to 1×10^7^ CFU ml^−1^. One hundred µl aliquots of the suspension were injected into the lateral tail veins of 4T1 tumor-bearing BALB/c mice 14 days after cell implantation. MRI of the tumor-bearing mice began 3 days after inoculation with bacteria. Following pre-induction scans, 200 µl of 25% L-arabinose was administered i.p. Mice were scanned 1 h and 24 h after arabinose administration. The mice were imaged under 1.5% Isoflurane anesthesia in a 7T small animal MRI machine. (GE Medical Systems MW) (TE/TR 10–80/4000 ms, Matrix 256×128, slice thickness 0.3 mm, Bandwidth 31.25, 3 averages.) T2 changes in the tumors were calculated using an in-house Matlab script and the T2 values were pseudocolored. Anatomical images were also acquired for the overlay using the same matrix. Imaging parameters: TE/TR 30/4000 ms, 6 averages.

### Localization of bacteria in colonized tumors by 3D image reconstruction

The data obtained from MRI were processed using ImageJ 1.37v and Amira V3.0. The tumor regions were manually segmented in each individual axial slice. The segmented data were used to generate three-dimensional surfaces. To visualize changes in the iron content within the tumor, the dark regions, presumably corresponding to the iron-loaded bacteria, were segmented automatically. Within each slice, all signal intensities from 0 to 40% of the mean grey level in the water phantom next to the tumor were selected. The water phantom was used as reference to eliminate variations in the signal intensities caused by the inhomogeneity of the coil. This approach was verified by comparing the mean grey level of the lightest region of the tumor with the mean grey level of the water phantom, showing that the ratios stay constant (data not shown). Low signal intensities at the tumor surface were omitted, since the bacteria are located at the border of necrotic and living tumor tissue within the tumor [6].

### Histology and fluorescence microscopy

Histology and fluorescence microscopy were performed essentially as described previously [6]. In brief, tumors were excised, snap-frozen in liquid N_2_, fixed, washed in PBS and embedded in 5% w/v low melt agarose (AppliChem, Darmstadt, Germany) in PBS.

Tissue sections were cut with a vibratome at a thickness of 100 µm and permeabilized in PBS containing 0.3% Triton X-100 for one hour. Sections were incubated for 12 to 15 h at room temperature with FITC-labeled Phalloidin (1∶200; Sigma, Taufkirchen, Germany) and biotinylated anti-*E.coli* antibodies (1∶200; Sigma, Taufkirchen, Germany). After several rinses with PBS, sections were incubated for 5 h with Cy3-labelled streptavidin, again rinsed with PBS, incubated for 1 h in 60% (v/v) glycerol in PBS, and mounted with 80% (v/v) glycerol in PBS.

Specimens were examined with the Stereo-Fluorescence microscope (MZ16 FA, Leica, Heerbrugg, Switzerland) that is equipped with a digital CCD camera (DC500, Leica). Digital images (1300×1030 pixel color images) were processed with Photoshop 7.0 (Adobe Systems) and merged to yield pseudo-colored pictures.

### Protein determination and iron uptake studies

For *in vitro* iron uptake studies, bacteria were grown at 37°C overnight in brain heart infusion broth (BHI), supplemented with 150 µM ferrous citrate and 100 µg ml^−1^ ampicillin. Twenty ml of overnight cultures were washed twice and resuspended in 20 ml ddH_2_O.

One ml of the suspension was concentrated tenfold and the protein content was determined using the Bradford assay (BioRad, Hercules, CA, USA). For SDS-PAGE, 10 µg protein samples were mixed with Laemmli buffer, incubated at 96°C for 5 min, then loaded onto a 15% SDS-polyacrylamide gel and electrophoresed at 200 mA prior to staining with Coomassie Blue. The percentage of ferritin-like proteins in the samples was determined using densitometry of the stained gels, which allowed the estimation of the quantity of ferritin-like molecules in the bacteria.

The remaining bacteria were harvested, resuspended in 2 ml ddH_2_O containing 10 µg Proteinase K, and incubated at 37°C for 24 h. Nitric acid (63%, 0.5 ml) was added to the cleared solution and the samples incubated on ice for 2 h. Following filtration through a 0.2 µm filter (Millipore), samples were analyzed by Inductively Coupled Plasma Mass Spectrometry (Varian, Darmstadt, Germany) to determine iron concentration as ^57^Fe against standard solutions (Merck, Darmstadt, Germany) of 500 and 1000 ppb Fe^2+^. Samples with higher concentration than 1000 ppb were diluted with double-distilled water to be within the calibration range.

Once the additional number of iron atoms in ferritin-like protein expressing strains compared to control strains was determined, the average number of iron ions within each holomer was calculated. For this calculation, an assumption was made that the additional iron was solely stored in the overexpressed proteins.

## Supporting Information

Figure S1 **T2 relaxation rate is dependent on the concentration of Bfr overexpressing** ***Ec*** **N.** Bfr overexpressing *Ec*N were diluted in control (GFP-expressing) *Ec*N and T2 relaxation rates were determined (Data represent mean (n = 3) +/− standard deviation of one representative experiment). T2 relaxation rates showed linear correlation (R^2^-value of 0.9759) with the concentration of Bfr-overexpressing *Ec*N.
(TIF)

Figure S2 **Three-dimensional reconstruction of the** ***Ec*** **N P_BAD_-** ***bfr*** **colonized tumor from** **Fig. 3** **before and 24 h after L-arabinose injection.** The surface of the tumor is transparent while dark voxels appear yellow (before L-arabinose injection) or red (after L-arabinose). The upper images show the 3D-reconstrcution of the tumor together with the plane that is shown in Fig. 3A. In the middle the same 3D-reconstruction is shown without the pictures from Fig. 3A. The lower images show the same tumor but from another angle.
(TIF)

Table S1 **Primer sequences used in this study.**
(DOCX)
