# Supplementary figures and images for: Magnetic Resonance Imaging of Tumors Colonized with Bacterial Ferritin-Expressing Escherichia coli
Source: PLoS One. 2011 Oct 3;6(10):e25409. doi: 10.1371/journal.pone.0025409 (PMC3184983; doi:10.1371/journal.pone.0025409)

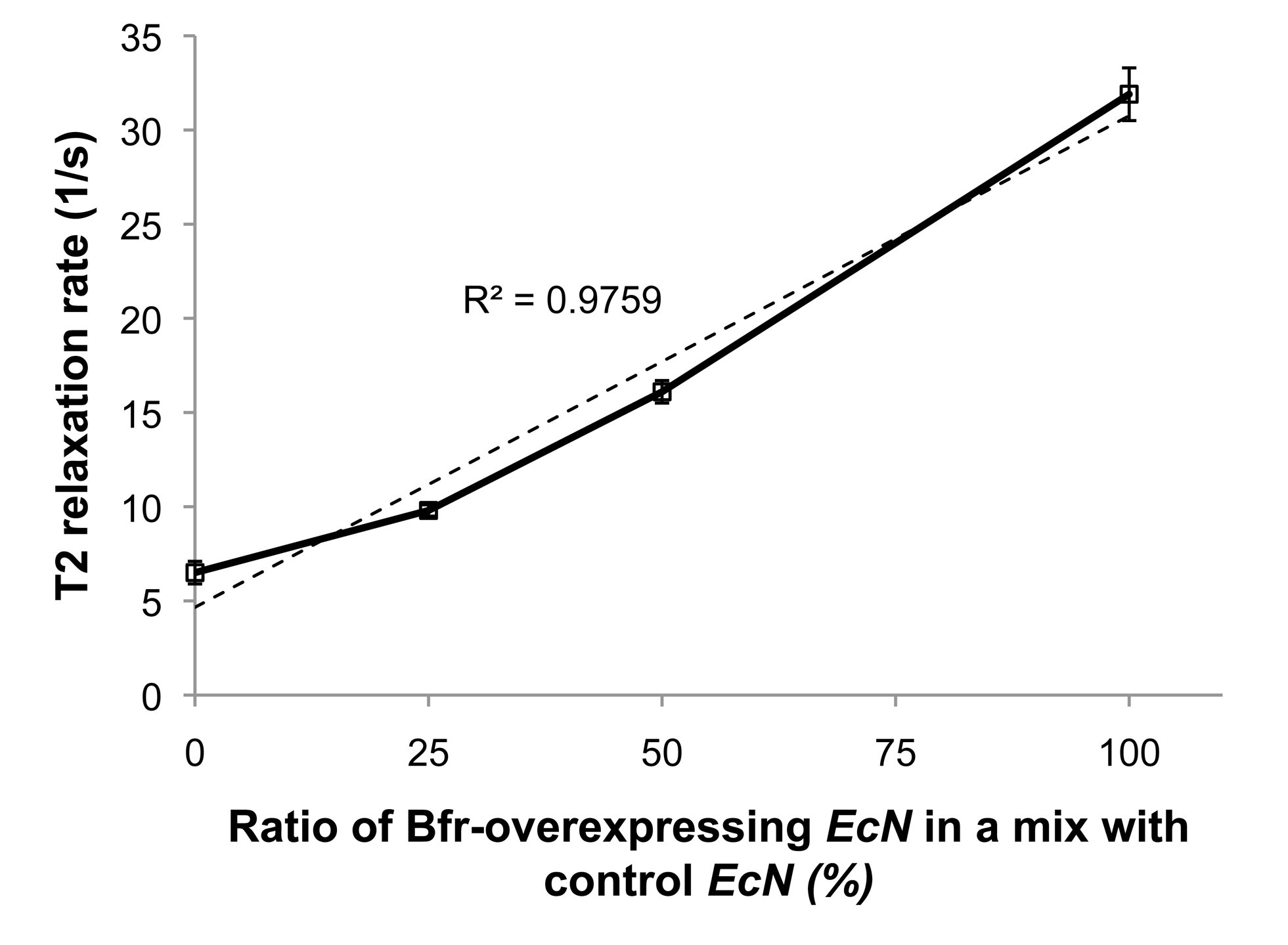

Supplement: Figure S1 — T2 relaxation rate is dependent on the concentration of Bfr overexpressing Ec N. Bfr overexpressing EcN were diluted in control (GFP-expressing) EcN and T2 relaxation rates were determined (Data represent mean (n = 3) +/− standard deviation of one representative experiment). T2 relaxation rates showed linear correlation (R2-value of 0.9759) with the concentration of Bfr-overexpressing EcN. (TIF) [file pone.0025409.s001.tif]

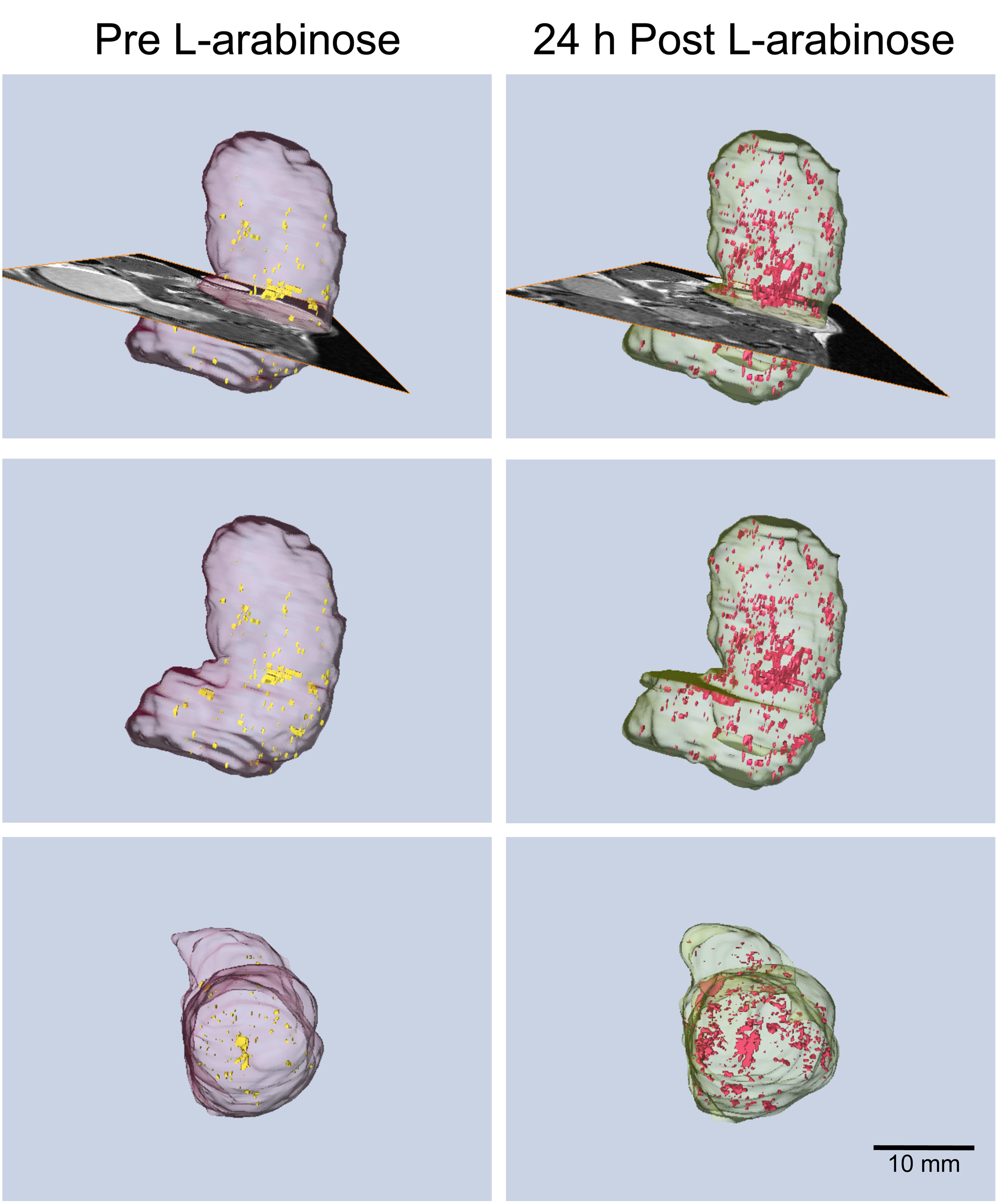

Supplement: Figure S2 — Three-dimensional reconstruction of the Ec N PBAD- bfr colonized tumor from Fig. 3 before and 24 h after L-arabinose injection. The surface of the tumor is transparent while dark voxels appear yellow (before L-arabinose injection) or red (after L-arabinose). The upper images show the 3D-reconstrcution of the tumor together with the plane that is shown in Fig. 3A. In the middle the same 3D-reconstruction is shown without the pictures from Fig. 3A. The lower images show the same tumor but from another angle. (TIF) [file pone.0025409.s002.tif]
