# Supplementary material for: Magnetic Resonance Imaging of Tumors Colonized with Bacterial Ferritin-Expressing Escherichia coli
Source: PLoS One. 2011 Oct 3;6(10):e25409. doi: 10.1371/journal.pone.0025409 (PMC3184983; doi:10.1371/journal.pone.0025409)
Supplement: Table S1 — Primer sequences used in this study. (DOCX) [file pone.0025409.s003.docx]

**Supplementary Table S1**: Primer sequences used in this study

| Name | Sequence (5’→3’) | Used for construction of: |
| --- | --- | --- |
| bfr-attB1F | ggggacaagtttgtacaaaaaagcaggcttataaaggaggaataaaaaatgaaag | pENTR-*bfr*_(M52L, M52H, L19P)_ |
| bfr-attB2R | ggggaccactttgtacaagaaagctgggtcaaccttcttcgcggatct | pENTR-*bfr*_(M52L, M52H, L19P)_ |
| bfr-M52L-for | atccattgatgagctgaaacacgccgatcg | pENTR-*bfr*_M52L_ |
| bfr-M52L-rev | cgatcggcgtgtttcagctcatcaatggat | pENTR-*bfr*_M52L_ |
| bfr-M52H-for | atccattgatgagcataaacacgccgatcg | pENTR-*bfr*_M52H_ |
| bfr-M52H-rev | cgatcggcgtgtttatgctcatcaatggat | pENTR-*bfr*_M52H_ |
| bfr-L19P-for | GTTGGGAAATGAGCCTGTCGCAATCAATCA | pENTR-*bfr*_L19P_ |
| bfr-L19P-rev | tgattgattgcgacaggctcatttcccaac | pENTR-*bfr*_L19P_ |
| ftn-attB1F | GGGGACAAGTTTGTACAAAAAAGCAGGCTAAGGAGGAATAAAAA ATG CTG AAA CCA GAA ATG ATT G | pENTR-*ftn* |
| ftn-attB2R | GGGGACCACTTTGTACAAGAAAGCTGGGTTTAGTTTTGTGTGTCGAGGG | pENTR-*ftn* |
| fri-attB1F | GGGGACAAGTTTGTACAAAAAAGCAGGCTAAGGAGGAATAAAAAatgaaaacaatcaactcagtagacacaaagg | pENTR-*fri* |
| fri-attB2R | GGGGACCACTTTGTACAAGAAAGCTGGGTctactctaatggagcttttcctaagaatgc | pENTR-*fri* |
| ccdBf ClaI | aaaaaaatcgatccagtgaattatcaactatgtataa | pBR322DEST and pBR322DEST_inv_ |
| ccdBr ClaI | aaaaaaatcgataccatgattacgccaagctatcaac | pBR322DEST and pBR322DEST_inv_ |
